# Chemically-Induced RAT Mesenchymal Stem Cells Adopt Molecular Properties of Neuronal-Like Cells but Do Not Have Basic Neuronal Functional Properties

**DOI:** 10.1371/journal.pone.0005222

**Published:** 2009-04-16

**Authors:** Gabriela F. Barnabé, Telma T. Schwindt, Maria E. Calcagnotto, Fabiana L. Motta, Gilberto Martinez, Allan C. de Oliveira, Leda M. N. Keim, Vânia D'Almeida, Rosália Mendez-Otero, Luiz E. Mello

**Affiliations:** 1 Departamento de Fisiologia, Universidade Federal de São Paulo (UNIFESP), São Paulo, São Paulo, Brazil; 2 Departamento de Biofísica, Universidade Federal de São Paulo (UNIFESP), São Paulo, São Paulo, Brazil; 3 Departamento de Pediatria, Universidade Federal de São Paulo (UNIFESP), São Paulo, São Paulo, Brazil; 4 Departamento de Biociências, Universidade Federal de São Paulo (UNIFESP), Santos, São Paulo, Brazil; 5 Instituto de Biofísica Carlos Chagas Filho, Universidade Federal do Rio de Janeiro (UFRJ), Rio de Janeiro, Rio de Janeiro, Brazil; Emory University, United States of America

## Abstract

Induction of adult rat bone marrow mesenchymal stem cells (MSC) by means of chemical compounds (β-mercaptoethanol, dimethyl sulfoxide and butylated hydroxyanizole) has been proposed to lead to neuronal transdifferentiation, and this protocol has been broadly used by several laboratories worldwide. Only a few hours of MSC chemical induction using this protocol is sufficient for the acquisition of neuronal-like morphology and neuronal protein expression. However, given that cell death is abundant, we hypothesize that, rather than true neuronal differentiation, this particular protocol leads to cellular toxic effects. We confirm that the induced cells with neuronal-like morphology positively stained for NF-200, S100, β-tubulin III, NSE and MAP-2 proteins. However, the morphological and molecular changes after chemical induction are also associated with an increase in the apoptosis of over 50% of the plated cells after 24 h. Moreover, increased intracellular cysteine after treatment indicates an impairment of redox circuitry during chemical induction, and *in vitro* electrophysiological recordings (patch-clamp) of the chemically induced MSC did not indicate neuronal properties as these cells do not exhibit Na^+^ or K^+^ currents and do not fire action potentials. Our findings suggest that a disruption of redox circuitry plays an important role in this specific chemical induction protocol, which might result in cytoskeletal alterations and loss of functional ion-gated channels followed by cell death. Despite the neuronal-like morphology and neural protein expression, induced rat bone marrow MSC do not have basic functional neuronal properties, although it is still plausible that other methods of induction and/or sources of MSC can achieve a successful neuronal differentiation *in vitro*.

## Introduction

Mesenchymal stem cells (MSC) from bone marrow stroma have been broadly studied for use in the therapy of neurological disorders. The great interest in studying this multipotent cell population largely derives from the advantages it offers. For instance, MSC can be easily harvested for autologous transplantation and do not involve ethical issues.

Many studies have demonstrated that MSC can give rise to neural cells, both *in vitro* [1]–[14] and *in vivo* [15]–[20]. *In vitro* experiments seeking the ideal conditions to obtain neural cells from MSC were initially conducted by two main research groups: Sanchez-Ramos et al. [6] and Woodbury et al. [9]. The former tried to differentiate MSC using molecules specifically involved in neural development (growth factors, neurotrophins, cytokines and retinoic acid) [6], while the latter proposed that neural induction could be performed simply by the addition of specific chemical compounds to the culture medium (β-mercaptoethanol [BME], dimethyl sulfoxide [DMSO] and butylated hydroxyanizole [BHA]) [9].

The chemical induction protocol used herein has two intriguing aspects: I) while the neuronal differentiation of embryonic stem cells and neural progenitor cells take days to happen [21], only a few hours are necessary for chemically-induced MSC to acquire morphological features and express specific markers of mature neurons (e.g., NeuN, MAP-2, NSE); and II) the percentage of differentiated neuronal cells exceeds 70%, which is extremely high compared to neuronal differentiation using growth factors [22]. Such results contradict the molecular mechanisms currently accepted for neural development because the development of mature neurons is a very slow process characterized by the induction and regulation of specific gene sets, as well as by distinct electrophysiological responses [23]–[25]. Thus, chemically-induced adult bone marrow MSC transdifferentiation is very unlikely to occur. Other reports suggest that neural differentiation induced by chemical compounds results from cytoplasmic retraction triggered by stress, which confers a neuronal-like morphology to the cells after treatment [26], [27]. However, these authors did not define the stressful conditions triggered by chemical induction. Despite the limited mechanistic data supporting this chemical induction method, it is still widely used by a considerable number of researchers [3], [5], [8], [28]–[30].

The objectives of present study are to evaluate the potential of a protocol based on chemical reagents in generating neurons from rat bone marrow MSC, and elucidate the electrical properties and molecular characteristics of these cells. Given that DMSO is an inhibitor of S-adenosylmethionine synthetase, an enzyme involved in homocysteine metabolism, and that BME is a potent disulfide bond reducer, changes in this metabolic route and redox state may be important determinants of cellular fate.

## Materials and Methods

### MSC Culture

Primary adult rat bone marrow MSC were isolated according to the method of Azizi et al. [17]. Briefly, Wistar adult male rats, 2 months old, were sacrificed in a CO_2_ chamber, according to the Animal Care and Use Committee of the Federal University of São Paulo. Tibias and femurs were dissected, the ends of the bones were cut, and 10 mL of DMEM (Gibco, Grand Island, NY, USA) were injected into the central canal of the bone to extrude the marrow. After Histo-Paque (density of 1.077 g mL^−1^) (Sigma, St. Louis, MO, USA) purification of whole bone marrow, mononuclear cells were isolated and cultured at a density of 2×10^5^ cells cm^−2^ in DMEM supplemented with 20% fetal bovine serum (Gibco) and 1% penicillin/streptomycin/amphotericin (Gibco). After 24 h, non-adherent cells were removed, and the medium was changed every 3–4 days until the culture became 80% confluent. The cells were tripsinized, split 1∶4 and passaged up to three times.

### Neuronal Cultures

Rat neural precursor cells (NPC) [31] and primary culture of rat fetal neurons from the brain cortex [32] were obtained as described previously [31], [32] (for details see supplementary data on file S1) and served as positive controls. NPC were used to simulate the differentiation of neurons from stem cells. Primary neuronal cultures were used as a common source of neurons.

### Fibroblast Culture

NIH 3T3 fibroblasts were maintained in DMEM containing 10% fetal bovine serum and 1% penicillin/streptomycin/amphotericin. These cells were used as negative controls in many experiments.

### Chemical Induction

Primary MSC were chemically induced to become neuronal-like cells *in vitro* according to the methods of Woodbury and colleagues [9]. Briefly, approximately 80% confluent MSC cultures were pre-induced for 24 h with DMEM, 20% fetal bovine serum, and 1 mM BME (Sigma). The pre-induction medium was then removed, and changed to serum-free induction medium consisting of DMEM containing 2% DMSO (Sigma) and 200 µM BHA (Sigma) for 4, 8 or 24 h.

### Control Groups

Matching MSC cultures were kept in standard medium (non-induced group, DMEM + 20% fetal bovine serum), or in serum-free condition (DMEM only) for 24 h, or were induced for 8 h and then returned to the standard medium overnight. NIH 3T3 fibroblasts, used as controls, were treated for 8 h with the chemical induction components (induced NIH 3T3), or kept in standard medium (non-induced NIH 3T3). The mononuclear fraction of bone marrow, after separation in Histo-Paque, was used in immunofluorescence and Western Blot assays. As positive controls for the neuronal differentiation protocol, NPC and primary neuronal culture were obtained as described above. Images of all groups were taken periodically with an inverted phase contrast microscope coupled to a digital camera (microscope model IX51-RC; Olympus, Japan) to record possible morphological changes in culture.

### Immunocytochemistry

NPC and all MSC groups were seeded into 24-well plates containing round coverslips. Chemically induced MSC were incubated in induction medium for 8 h. After treatment, cells were fixed with 4% paraformaldehyde in 0.1 M PBS (pH 7.2) for 30 minutes and processed for immunocytochemistry to assess for cell differentiation, as described below. Nonspecific antibody reactions were blocked with 5% normal goat serum (Gibco) for 1 h at room temperature and cells were permeabilized with 0.1% Triton X-100 (Sigma). Next, cells were incubated overnight at 4°C with primary antibodies diluted in blocking solution directed against GFAP (Dako, Denmark, 1∶500), NeuN (Chemicon, Temecula, CA, USA, 1∶500), β-tubulin III (Sigma, 1∶500), MAP-2 (Chemicon, 1∶250), NSE (Dako, 1∶200), NF-200 (Sigma, 1∶100) and S100 (Sigma, 1∶100). After three washes with PBS, cells were incubated with the respective anti-mouse or anti-rabbit biotinylated secondary antibodies (1∶200; Vector Laboratories, Burlingame, CA, USA) for 1 h at room temperature, followed by 1 h of incubation in avidin-biotin peroxidase complex (1∶100; Vector Elite Kit) at room temperature. Diaminobenzidine (0.05%; Sigma) with nickel chloride (0.04%) was used as chromogen, with reactions sustained for 10 minutes at room temperature. Cells were then coverslipped with Entellan (Merck, Germany) on glass slides. NPC were used as positive controls for immunolabeling; for negative control primary antibodies were omitted (figure S1). Cells were analyzed under a bright field microscope (Nikon Eclipse E600 FN, Nikon, Japan) and images were captured by a coupled camera system using ACT-1 software (v.2.2, Nikon). This experiment was performed independently for three times using two coverslips of plated cells for each marker (n = 6). Cells were counted and the proportion of positive stained cells was estimated by dividing the number of positive cells by the total cells number in the same field. Statistical analysis consisted of ANOVA (95% of confidence interval) followed by Newman-Keuls post-hoc test.

### Immunofluorescence

The bone marrow mononuclear cell fraction of 5 adult rats (3 to 6 months old) was obtained after separation of total bone marrow in Histo-Paque, as described above. Cells were immediately fixed in 1% paraformaldehyde and distributed on gelatinized glass slides by cytocentrifugation at a density of 5×10^5^ cells mL^−1^ in 200 µL per slide.

Cells were washed and incubated in blocking solution with primary antibodies against GFAP, β-tubulin III, NeuN, NSE, NF-200 and S100, followed by fluorescent secondary antibodies (Cy3 conjugated; Jackson Immuno Research, West Grove, PA, USA, 1∶1000), in the same conditions as described for immunocytochemistry. Nuclei were counterstained with DAPI and slides were coverslipped with paraphenylenediamine. Cell images were taken by Axiocam MRm camera system (Carl Zeiss Inc., Thornwood, NY, USA) coupled to a fluorescent microscope (Axiovert 200M, Carl Zeiss Inc.). Positive staining and total cells (2,000 cells for each marker) were counted and analyzed as described in the immunocytochemistry method.

### Western Blot

Cell extracts were obtained with RIPA buffer in the presence of protease inhibitors (sodium pyrophosphate, sodium fluoride, phenylmethanesulfonyl fluoride, pepstatin A, aprotinin, leupeptin, antipain, benzamidine - all from Sigma). Eighty micrograms of protein extract from MSC (non-induced, serum-free and 8 h chemically-induced groups), bone marrow mononuclear cells, NIH 3T3 (non-induced and chemically-induced) and adult rat brain cortex were electrophoretically separated in a 10% acrylamide gel and transferred onto a nitrocellulose membrane (GE HealthCare, England). The blot was probed for NeuN (Chemicon, 1∶1000), NF-200 (Sigma, 1∶500), β-tubulin III (Sigma, 1∶1000), stripped, and probed for actin (Chemicon, 1∶2000). Secondary antibodies were horseradish peroxidase conjugated (HRP, Santa Cruz Biotechnology, Santa Cruz, CA, USA) and detected with ECL reagents (GE HealthCare, England). Light-sensitive silver radiographic films (HyperFilm, GE HealthCare, Sweden) were exposed to ECL and developed.

### Flow Cytometry

Non-induced, serum-free and chemically-induced MSC were evaluated for cell death during the induction protocol, using Annexin V and propidium iodide (PI) as markers for apoptosis or necrosis, respectively (Annexin V-FITC Apoptosis Detection Kit I; BD Biosciences, San Diego, CA, USA). The chemically-induced MSC group was divided into 4 subgroups: BME, where cells were treated only with BME for 24 h; 24 h BME treated cells induced with the DMSO/BHA mix for 4, 8 and 24 h. Adherent cells were trypsinized and collected in a tube, followed by centrifugation to remove trypsin and medium. Cells were then washed in PBS twice and resuspended in 100 µL of Binding Buffer (BD Biosciences). Subsequenlty, 5 µL of anti-Annexin V antibody and 10 µL of PI were added to each tube, and cells were incubated at room temperature for 15 minutes. MSC from the non-induced group were used to set the cytometer parameters: 1) without Annexin V and PI; 2) Annexin V only; 3) PI only. Cell counts were taken in a BD FACSCalibur (BD Biosciences) and analyzed with Cell Quest Pro software (BD Biosciences). The following parameters were used: FL1 for Annexin V, FL2 for PI, side scatter and forward scatter. The experiment was made in triplicates (n = 3) and 30,000 events were registered for each sample. Statistical analyses were performed using ANOVA with Newman-Keuls post-hoc test. Significance level was set at *p*<0.05.

### Homocysteine and cysteine quantification

Non-induced and 8 h chemically-induced MSC cultures (n = 9, each group) were used for biochemical analysis. Cells were washed 3 times in PBS, and detached from the plate surface with cell scrapers in the presence of protease inhibitors (sodium pyrophosphate, sodium fluoride, phenylmethanesulfonyl fluoride, pepstatin A, aprotinin, leupeptin, antipain, benzamidine - all from Sigma) diluted in PBS. Cells were collected in 2 mL cryovial tubes and submitted to five freeze and thaw cycles, by alternating between dry ice and water bath at 37°C. Cell lysates were stored at −80°C until chemical analysis.

Intracellular Hcy and Cys were measured by high performance liquid chromathography with fluorimetric detection and isocratic elution. This methodology was adapted from Pfeiffer et al [33] and involves three steps, namely reduction of thiol groups using TCEP (tris(CarboxyEthyl)Phosphine), protein precipitation with trichloroacetic acid, and derivatization with SBD-F (7-fluorobenzene-2-oxy-1,3-diazolic-4-ammonium sulfate). The Shimadzu high performance liquid chromathography system had a SIL-10ADvp automatic sample injector and a RF-10AXL fluorescence detector. Chromatographic separation was performed using a C18 model Shim-pack CLC-ODS column (4.6×150 mm, with 5.0 µm micro particles). The fluorescence was detected at a 385 nm excitation and at a 515 nm emission. Total Hcy and Cys levels were measured using a calibration curve of defined Hcy and Cys concentrations with cystamine as the internal standard normalized to the total protein content of the cell extract. Due to inter-assay variation, total Hcy and Cys levels were expressed as the ratio of each measure to the mean content of the control values. Statistical analyses were performed using *t*-Student test with significance level set at *p*<0.05.

### Electrophysiology

*In vitro* patch clamp recordings were used to distinguish chemically-induced MSC from neurons by their electrophysiological features, such as action potential firing properties, presence of K^+^ and Na^+^ currents, and presence of voltage-gated channels. Neurons from primary culture of E17 cortex and standard cultured MSC were used as controls. The cell-attached voltage-clamp and whole-cell current-clamp or voltage-clamp configurations were performed in cells placed in a bath solution consisting of (in mM) 137 NaCl, 2.7 KCl, 1.36 NaH_2_PO_4_, 0.49 MgCl_2_ 6H_2_O, 11.9 NaHCO_3_, 1.36 CaCl_2_2 H_2_O, and 5.04 Glucose, and with a pH of 7.4 (Tyrode). Whole-cell pipette recordings were obtained from either MSC or neurons visually identified using a microscopy system (model Diaphot, Nikon). Patch electrodes (3–7 MΩ for whole cell or 8–15 MΩ for cell-attached) were pulled from 1.5 mm o.d. borosylicate glass capillary tubing using a micropipette puller. For whole-cell K^+^ current recordings, the intracellular solution contained (in mM) 1 CaCl_2_, 140 KCl, 1 MgCl_2_, 10 HEPES, 10 EGTA, 2 Na_2_ATP and 0.2 Na_2_GTP, and had a pH of 7.2. To study the K^+^ current, the holding membrane potential was −50 mV. The voltage-clamp protocol consisted of 100-ms depolarizing steps with 10-mV increments at 0.5 Hz, taking the membrane from −60 to +60 mV. For whole-cell Na^+^ current recordings, intracellular solution consisted of (in mM) 140 CsCl, 2 K-ATP, 4 MgCl_2_, 10 EGTA and 10 HEPES, had a pH of 7.3, and 300 Osm. Moreover, 4-aminopyridine (5 mM) and tetraethylamonium (10 mM) were added to the Tyrode extracellular solution to block K^+^ currents. The voltage-clamp protocol to study the Na^+^ current consisted of a 100-ms hyperpolarizing pulse from a holding potential of −80 to −120 mV to remove channel inactivation, followed by 200-ms depolarizing steps with 10-mV increments at 0.5 Hz, bringing the membrane from −80 to +70 mV.

Intracellular patch pipette solution for whole-cell current-clamp recordings contained (in mM) 120 K-gluconate, 10 KCl, 1 MgCl_2_, 0.025 CaCl_2_, 0.2 EGTA, 2 Na_2_-ATP, 0.2 Na_2_-GTP and 10 HEPES, had a pH of 7.2, and 295 Osm. In current-clamp, current pulses (50–1000-ms, 0.5 Hz) were given through the patch pipette to examine whether the cells were capable of producing action potentials.

Single-channel currents were recorded using the cell-attached mode of the patch-clamp technique. Cell-attached patches were performed in the presence of high-KCl solution, both in the bath and pipette, which contained (in mM) 150 KCl, 1 MgCl_2_.6H_2_O, and 10 HEPES, and had a pH of 7.4. Patches were examined at a holding potential of +60 mV. After a patch was obtained, a stabilization period of ∼5 minutes was allowed; patches that showed large fluctuation in channel activity over this period were discarded. Thereafter, channel activity was recorded continuously until the end of the experiment. The duration and amplitude of each current level were determined using idealized records from the original data, constructed through the recognition of the transitions between distinct levels. *NP*_o_ values, where *N* is the number of channels in the patch available to open and *P*_o_ is the open probability of the channel, were calculated by the ratio of the mean current to the unitary single-channel current. The mean current was obtained from the amplitude current distribution histogram, using the following expression: *I*_mean_ = A_1_f_1_+A_2_f_2_+..... A*_n_*f*_n_* where A_1_, A_2_, and A*_n_* represent the area under the Gaussian curve for each current level (f_1_, f_2_, f*_n_*) present in the patch.

Voltage and current were registered with the Axopatch 200 amplifier (Axon Instruments/Molecular Devices Corp., Union City, CA). Whole-cell voltage-clamp data were filtered at 1 kHz and cell-attached data were filtered at 5 kHz (3 dB, 8-pole Bessel filter), and monitored with pClamp 6.0 software (Axon Instruments/Molecular Devices Corp.). The resulting currents and voltage were analyzed using Clampfit 9.0 (Axon Instruments/Molecular Devices Corp.).

### Ethical issues

This work was developed under the approval of the Ethics Committee of Federal University of São Paulo (UNIFESP), process 01093-06.

## Results

### Acquisition of neuronal-like morphology by MSC subjected to chemical induction is due to retraction of cytoplasmic processes

**Figure 1:**
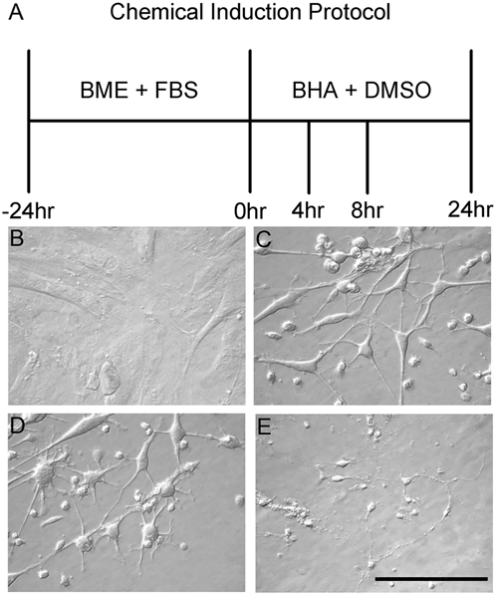
Morphological comparisons between non-induced MSC, chemically-induced MSC, chemically-induced NIH 3T3 and primary neurons. A) Schematic illustration of the chemical neuronal induction protocol. B–D) Phase-contrast microscopy images showing morphological aspects. B) MSC have a spread-out cytoplasm in standard medium; C) after induction for 8 h cells exhibited neuronal-like morphology; D) fibroblast NIH 3T3 after 8 h induction adopted neuronal-like morphology; E) primary neuronal cultures. Scale bar = 100 µm.

Adult rat bone marrow-derived MSC were isolated and successfully expanded, displaying characteristic morphology and colony formation [34]. Morphological alterations were observed a few hours after MSC induction following the protocol proposed by Woodbury et al. [9] (figure 1), thus reproducing the effects described previously. Those cells showed morphological similarities to neurons. Changes in cell shape were gradual and due to retraction of cytoplasmic processes. A long period of induction (24 h) caused complete processes retraction and cells acquired a round shape, leading to detachment from the culture plate surface. To evaluate cell death we performed Annexin V and PI staining, as demonstrated below. To properly evaluate cell death and detachment, experiments were done at 8 h post-induction, when 80% of the initial amount of cells was likely to be alive and 82% displayed a neuronal-like shape (figure 1).

**Figure 2:**
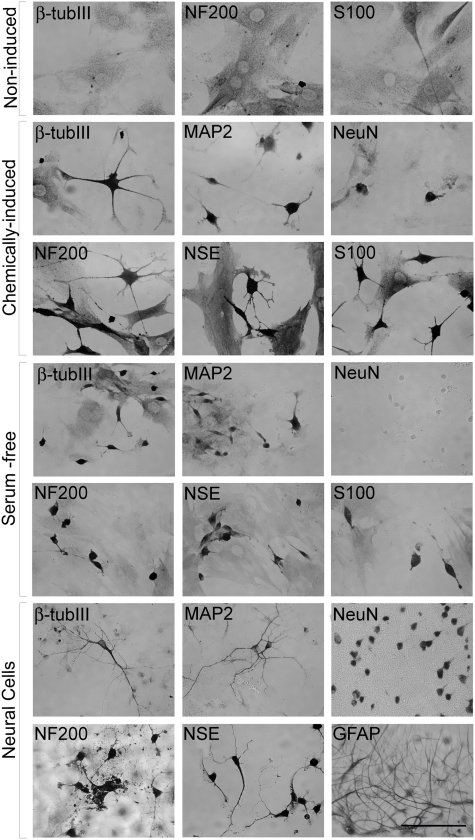
This panel shows the pattern of neural markers expression in the non-induced, chemically-induced and serum-free MSC groups. Primary neuronal cultures were used as positive controls for the immunocytochemistry. Phase microscopy images of cells stained for: β-tubulin III (β-tubIII), MAP-2, NeuN, NF-200, NSE, S100 and GFAP. Scale bar = 100 µm.

Acquisition of neuron-like morphology also occurred for MSC cultured in the absence of fetal bovine serum for 24 h (likely to represent a stressful condition) (figure 2). After chemical induction and re-exposure to fetal bovine serum, cells reacquired their typical normal morphology, with a spread-out cytoplasm, and there were no longer cells with neuronal-like morphology (data not shown). As demonstrated in figure 1D, NIH 3T3 fibroblasts also adopted neuronal phenotype after exposure to the chemical induction protocol. Our data suggest that MSC display morphological alterations as a response to the chemical compounds used for the induction of neuronal fate or to the lack of normal supporting substances (such as fetal bovine serum).

### Expression of neural proteins is not restricted to chemically-induced MSC

Immunocytochemistry results showed that induced cells with neuronal-like morphology were positively stained for NF-200, S100, β-tubulin III, NSE and MAP-2 proteins (figure 2). MSC in the serum-free condition also stained for all the markers that appeared in the chemical induction group, except for NeuN. NeuN immunostaining of chemically-induced MSC had low intensity compared to neurons from rat brain section (figure 2). Cell staining was found at a similar intensity level in the entire cytoplasmic extension of induced MSC as well as in their extension processes, while the localization of these proteins is normally restricted to specific neuronal regions (dendrites: β-tubulin III; axons: MAP-2). Culture of neural cells expressed all of the expected markers including GFAP that did not appear in any of the other MSC culture conditions.

**Figure 3:**
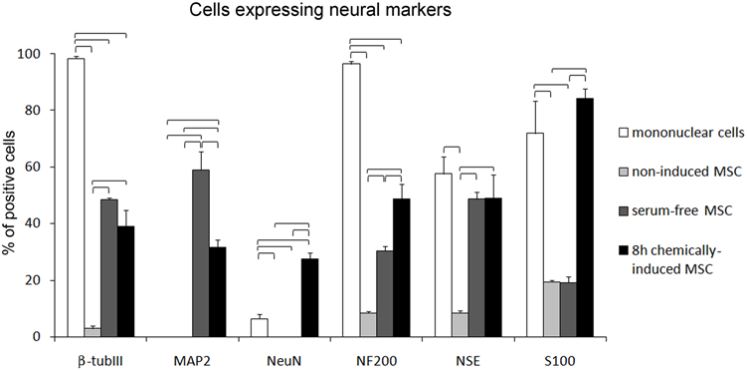
Proportion of positive stained cells for neural proteins in rat bone marrow mononuclear cells and in non-induced, serum-free and chemically-induced MSC. For each neural marker, statistically significant differences (ANOVA, p<0.05) between cell types/conditions appears under brackets.

The counting of immunostained cells in bone marrow mononuclear fraction, non-induced, serum-free and chemically-induced MSC shows the gene expression profile of neural markers (figure 3). All other neural markers used in this work were found in mononuclear cell fraction, except MAP-2. In standard culture conditions, MSC (non-induced) did not express MAP-2 and NeuN, but a small fraction of cells were positive for β-tubulin III, NF-200 and S100. The proportion of positive staining increased in serum-free condition for β-tubulin III, MAP-2, NF-200 and NSE, when compared to non-induced MSC. Finally, chemical induction always had a higher proportion of positive cells than non-induced, with a remarkable increase in the percentage of NeuN, NF-200 and S100 staining compared to the serum-free condition.

**Figure 4:**
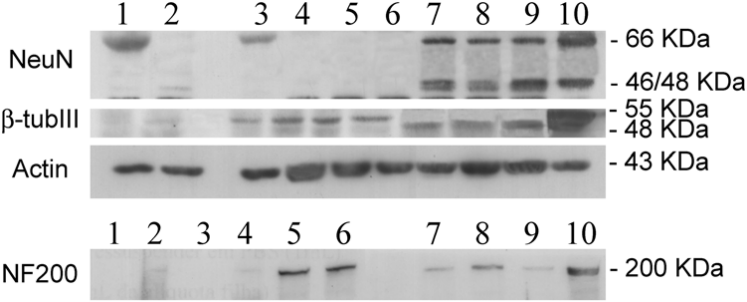
Analysis of the expression of NeuN, NF-200 and β-tubulin III by Western Blot. Lanes: 1) non-induced NIH 3T3; 2) chemically-induced 3T3; 3) non-induced MSC; 4) serum-free MSC; 5 and 6) chemically-induced MSC for 8 h; 7, 8 and 9) freshly extracted bone marrow mononuclear cells; 10) brain tissue (positive control). Actin (43 KDa) was used as an internal control.

Considering the Western Blot analysis (figure 4), samples from non-induced NIH 3T3 (lane 1) and non-induced MSC (lane 3) expressed only the 66 KDa NeuN isoform, while bone marrow mononuclear cells (lanes 7, 8, 9) and brain (lane 10) extracts had all three NeuN isoforms (66, 46, 48 KDa). The antibody against β-tubulin III cross reacts with the rat tubulin, and explained the 55 KDa band. Only bone marrow mononuclear cells (lanes 7, 8, 9) and brain (lane 10) samples had the 48 KDa band corresponding to the β-tubulin III expression. NF-200 expression increased after MSC chemical induction (lanes 5, 6) and is also present in bone marrow mononuclear cells (lanes 7, 8, 9). A small increase in NF-200 expression was observed after serum deprivation of NIH 3T3 cells (lane 2) and of MSC (lane 4). Actin (43 KDa) was used as an internal control.

### Chemical induction leads to apoptosis

**Figure 5:**
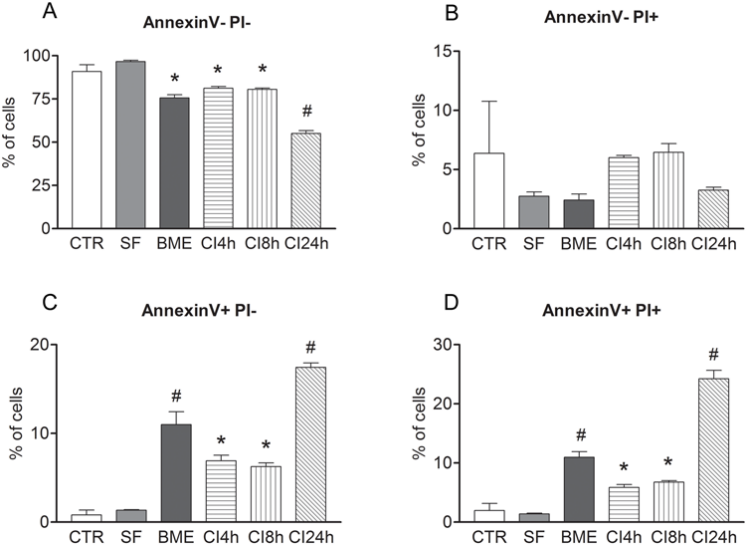
Cell death analysis of MSC. Graphic representations of the percentage of MSC that are: A) live cells (Annexin− PI−); B) necrotic (Annexin− PI+); C) early apoptotic (Annexin+ PI−); or D) late apoptotic (Annexin+ PI+) in the non-induced (control, CTR), serum-free (SF), pre-induced (BME) or chemically-induced (for CI4 h, CI8 h and CI24 h). Mean±standard error. p<0.05, # different from every other group; * different from non-induced and serum-free.

Flow cytometry analysis for Annexin V and PI indicated that during the chemical induction process there was an increase in cell death (figure 5). Although serum deprivation for 24 h is characterized as a stressful condition for MSC, it was not sufficient to trigger cell death mechanisms. Furthermore, serum-free MSC show the same proportions of live and dead cells as non-induced MSC. At the end of the chemical induction protocol (24 h) we found a significant percentage of dead cells (44.8%; figure 4A; ANOVA *p*<0.05, followed by Newman-Keuls as compared to every other group, *p*<0.01). Our results also demonstrated that most of cells died due to apoptotic events during chemical induction. Indeed, Annexin V^−^/PI^+^ cells, likely to indicate necrotic elements, were less frequent than Annexin V^+^ (both PI^+^ and PI^−^) likely to indicate apoptotic events. Putative early apoptotic cells (Annexin V^+^/PI^−^) increased after exposure to BME and BHA/DMSO at 4 h, and were augmented after exposure to BHA/DMSO at later times with the highest value occurring at 24 h (ANOVA *p*<0.05, followed by Newman-Keuls all groups as compared to non-induced group, and chemically-induced for 24h as compared to every other group, *p*<0.01; figure 4C). The number of late apoptotic cells (figure 4D) significantly increased after BME pre-induction treatment (11%) as compared to non-induced MSC and at the 24 h induction time point (24.2%) as compared to every other group. We have thus found a number of significant differences between non-induced and chemically-induced MSC groups for live and apoptotic cells (early or late apoptotic; figure 5).

### High demand over redox circuitry during chemical induction

**Figure 6:**
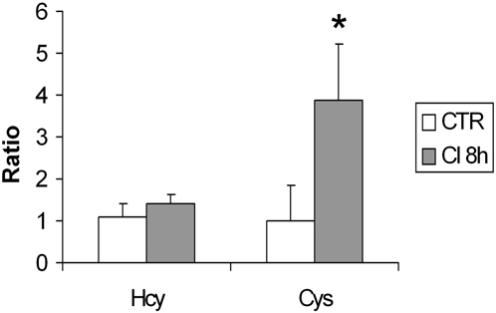
Ratio between Hcy e Cys contents obtained for non-induced (control, CTR) and chemically-induced MSC. No cellular change in Hcy content was observed in the induction protocol (p = 0.23). Total Cys content increased almost four times (p = 0.00006) in chemically-induced MSC compared to non-induced ones. Data represented as mean±standard deviation.

In order to evaluate whether the chemical compounds used in the neuronal induction could promote alterations in the redox balance, we analyzed the Cys and Hcy cellular contents. No cellular change in Hcy content was observed in chemically-induced MSC. However, total Cys content increased almost four times in induced MSC as compared to non-induced cells (figure 6).

### Neuronal-like MSC do not exhibit neuronal electrical properties

**Figure 7:**
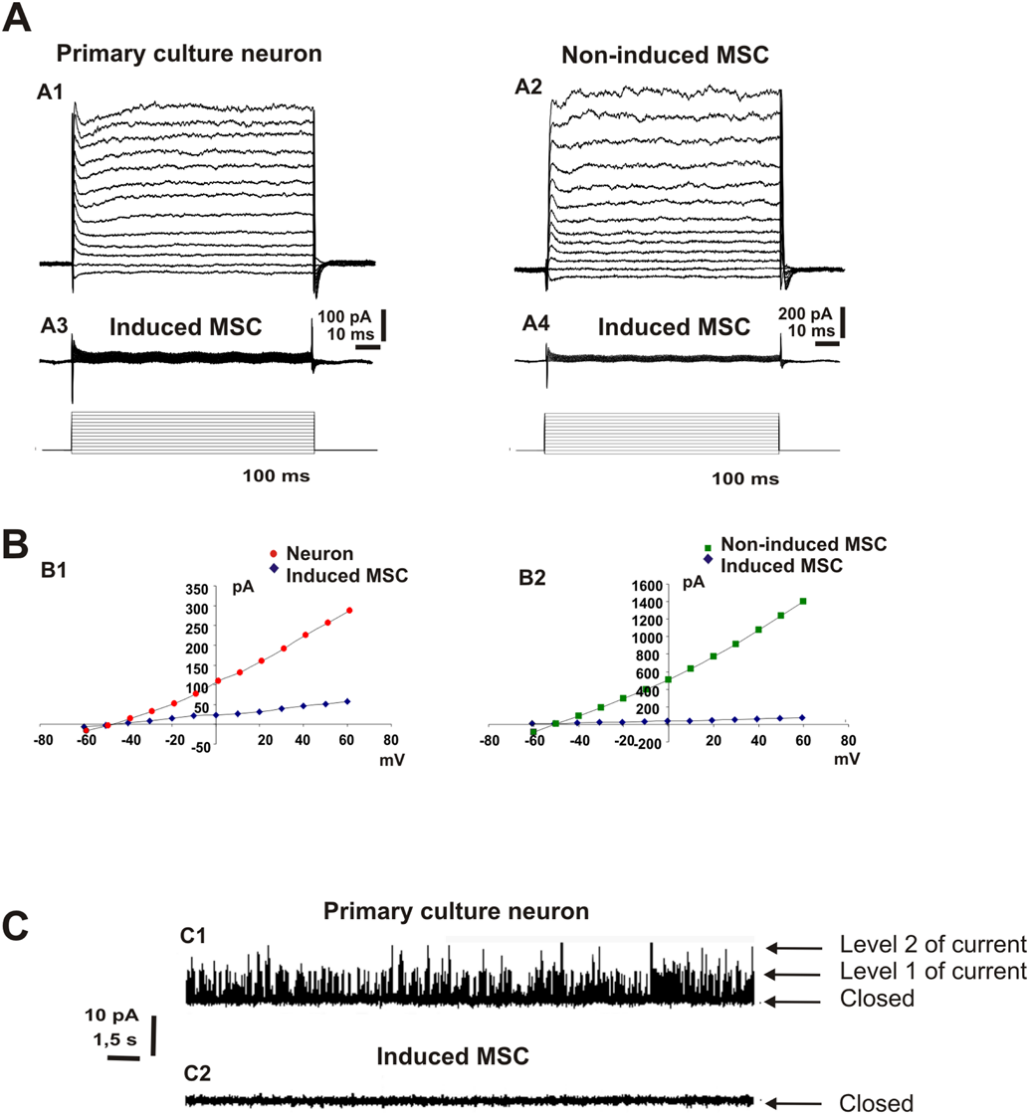
Electrophysiological recordings of K^+^ current. A) Voltage-dependent K^+^ current recorded from primary culture neuron (A1), non-induced MSC (A2) and chemically-induced MSC (A3 and A4). B) I/V curves calculated from traces in A showing decreased K^+^ current for treated MSC (B1, B2). C) Single-channel K^+^ current recordings showing frequent channel opening in primary culture neurons (C1) but absent in chemically-induced MSC (C2).

**Figure 8:**
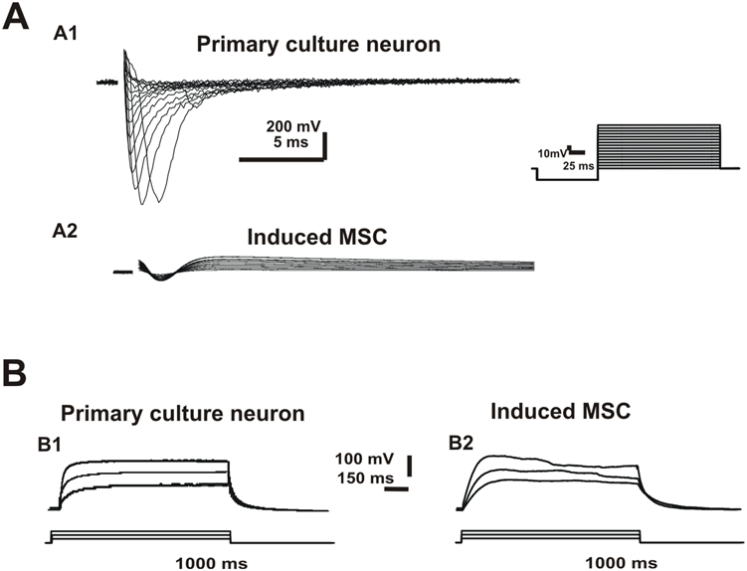
Electrophysiological recordings of Na^+^ current and AP firing ability. A) Representative voltage-dependent Na^+^ current recorded during a 100-ms hyperpolarizing pulse, followed by 200-ms depolarizing steps (10-mV increments) (see inset) for primary culture neuron (A1), and chemically-induced MSC (A2). B) Membrane potential of chemically-induced MSC recorded under current clamp. Note the inability to fire AP with depolarizing current steps (50 or 1000 ms).

To determine whether neuronal-like cells derived from adult rat MSC were electrically active, we performed patch-clamp recordings to study the presence of K^+^ and Na^+^ currents (whole-cell voltage-clamp), voltage-gated channels (cell attached) and the ability of cells to fire action potentials (whole-cell current-clamp). We observed that both neurons from E17 cortex primary culture (n = 6) and untreated MSC (n = 5) exhibited K^+^ currents (Figue 6 A1, A2, B1, B2). However no K^+^ current was detected from chemically-induced MSC with neuronal-like morphology (n = 7) (figure 7 A3, A4, B1, B2). These cells also did not present Na^+^ current when compared with controls (neurons from primary culture n = 6; chemically-induced MSC n = 10) (figure 8 A1, A2). We observed that both, the immature neurons from primary culture and the chemically-induced MSC were unable to fire action potentials when depolarizing currents were injected in current-clamp recordings (n = 3; n = 5) (figure 8 B).

Lastly, cell-attached voltage-clamp recordings were performed to evaluate the presence of voltage-gated channels in the cell membrane (figure 7 C). Here again, MSC subjected to the chemical treatment differed from embryonic rat neurons. The activation of K^+^ channels in response to membrane depolarization (+60 mV) occurred only in primary cultured neurons (*NP*_o_ = 0.04) (n = 3) (figure 7 C1), but no activation was observed for chemically-induced MSC (*NP*_o_ = 0) (n = 5) (figure 7 C2).

## Discussion

The potential of adult bone marrow MSC to become neurons is controversial. When cells are removed from their niche and propagated *in vitro*, they lose many of their original properties (proliferation rates, gene expression, morphology, cell-cell interaction) and can behave in a non-physiological way, leading to artifacts [35]. Alternatively, neurons derived from bone marrow stromal cells after transplantation could be explained by cell fusion, giving rise to uncertainties over “real” transdifferentiation [36], [37]. In this context, the demonstration of whether or not MSC can differentiate into neurons is not unequivocal. Herein we emphasize that morphological and gene expression alterations should be accompanied by electrophysiological demonstrations that MSC derived neurons are functional cells.

The main findings of this work are summarized: i) the seeming induction triggered by one specific protocol based on chemical compounds is reversible and caused by cytoplasmic retraction due to stress; ii) adult rat bone marrow mononuclear cells also express some neural markers irrespective of being subject to chemical induction; iii) apoptosis rates are increased over induction time; iv) redox circuitry is under high demand during chemical induction; v) adult rat bone marrow MSC using this specific neuronal induction protocol do not present electrical properties of fetal neurons.

It was previously described that some bone marrow cell populations, including MSC, express neural markers even before any neural induction protocol [3], [38]–[42]. These data are in agreement with our findings for adult bone marrow mononuclear cells, where we found S100, β-tubulin III, NeuN, NSE and NF-200 expression by immunocytochemistry and/or Western Blot analysis. It has been suggested that an overlap between the genetic programs of bone marrow adult stem cells and neural stem cells [38], [42] exists, but this does not mean that the same proteins play the same roles in different cell types.

NRSF/REST mediates the transcription repression of neuron-specific genes in non-neuronal cells [43], [44]. The transcriptional repressor neuron-restrictive silencer factor has a critical role in the specific control of connexin36 in insulin-producing cell lines, indicating that connexin36 participates to the neuronal phenotype of the pancreatic beta-cells [45]. In order to explain the presence of neural markers in bone marrow mononuclear cells, we can hypothesize that neuronal gene expression in a specific subpopulation could be due to release of neuron-restrictive silencer element.

When bone marrow mononuclear cells were plated for *in vitro* expansion, a subpopulation was selected by the property of adhesion to plastic surface. The resulting adherent MSC present low levels or do not express any of the tested neural proteins. Whether this is a feature of this subpopulation or cells down-regulate neural protein expression after plating is still unknown [46]. But when fetal bovine serum was removed or cells were chemically induced with specific compounds, the expression of these proteins became high. In fact, immunocytochemistry experiments showed that cells with neuronal-like morphology express neuronal proteins. Other groups also have demonstrated that chemical induction triggers the increase in the transcription and expression of some genes that code for neuronal proteins [9], [27], as well as serum-free media, when fetal bovine serum is removed [35]. As we observed, this increased gene transcription/expression may be related to stressful factors [26]. For example, NSE is increased after brain injury and in cancers [47].

Measures of cell death during this specific induction process are indicative of toxicity of the chemical compounds used. Here we show that most cells die within 24 h of chemical induction, in agreement with other studies [29]. We can consider a gradient of cellular stress in which the mild fraction triggers the expression of genes that might help cells to survive; and a severe, long-lasting cellular stress culminates with cell death. Here we can compare the mild stress to the serum-free media, and the severe to the chemical induction. In this way, it is possible we had found less positive cells for some neural markers after chemical induction than in serum-free MSC because the stained cells already died after 8h induction. Accordingly, the toxic environment should trigger abnormal responses in the cells, far from what would constitute a physiological response.

Altered total cysteine content in these cells can also be interpreted in this context. In the presence of a S-adenosylmethionine synthetase inhibitor, i.e., DMSO, the increase in cysteine content must be related to increased cysteine uptake. Cells are able to take cysteine from the culture media both in its oxidized, cystine, and reduced forms. Under the condition of an intracellular reduced state determined by BME + BHA, cystine uptake is most likely a compensatory mechanism to maintain the adequate redox potential in these cells. Cystine is the most oxidized intracellular thiol/disulfide couple, and such a situation may have homeostatic purposes as it can serve as an oxidant signal to control normal cell function [48].

It is possible that redox modulation can drive neuronal differentiation, but non-physiological changes are more likely related to cell damage [49], [50], [51]. A recent work demonstrated that MSC treated with reduced glutathione apparently differentiated into neurons [52]. The induction protocol in this paper used serum-free culture medium, which we demonstrated to be sufficient to induce the morphological changes in MSC formerly believed to be neurons.

The electrophysiological data indicate that chemically-induced cells with neuronal phenotype do not exhibit intrinsic properties of neurons. The absence of action potential suggests that either the cells are immature neurons or they are not neurons. Recently, using a protocol described by Woodbury et al. [9], Wenisch et al. [30] studied the intrinsic properties of the induced MSC and showed that they were unable to fire spontaneously or evoke action potential. However, Wenisch et al. [30] concluded that these cells could be immature neurons, based on some ultra-structural characteristics similar to that of neurons.

Although immature neurons may not fire an action potential, they exhibit ion voltage-gated channels producing K^+^ and Na^+^ currents in response to depolarization. The membrane permeability to K^+^ and Na^+^ increases during depolarization of excitable cells [53] and currents could be identified using the *voltage-clamp* technique. According to our results, chemically-induced MSC from adult rat bone marrow did not present K^+^ or Na^+^ currents (in contrast to what was recorded in immature neurons). Moreover, chemically-induced MSC are electrophysiologically different from fibroblasts, since they present a class of maxi K^+^ channels [54].

An interesting observation is that non-induced bone marrow MSC have voltage-gated K^+^ and Na^+^ channels [55]. But when these cells are chemically induced they lost this characteristic. It is possible that the redox imbalance caused by the chemical compounds interfere in MSC ion channel function/activation. Some ion channels are modulated or modified by oxidative stress, resulting in modified ion conductance (K^+^, Ca^2+^ or Na^+^) [49], [50]. The abnormal function of these ion channels can alter the membrane potential. Moreover, increased concentration of intracellular Ca^+^ can trigger apoptosis and K^+^ efflux causes cell shrinkage [56]. Thus, cell shrinkage, altered activity of ion channels and cell death are all closely related phenomena [57]. Following MSC chemical induction, we could clearly notice the interplay between these three events.

Holistically, our data allow us to suggest that bone marrow MSC are damaged in the presence of toxic substances that constitute one specific chemical induction protocol and, consequently, present abnormal responses. The expression of neural proteins could be one of these abnormalities that may not be necessary (e.g. adult Purkinje cells do not express NSE protein in their somas) [58], and is definitely not sufficient (current results) to classify any single cell as a neuron. Indeed, even the most distinctive neuronal feature, i.e., the ability to fire action potentials, is not present in every single neuron (e.g., bipolar retinal cells lack this capacity). The development of the nervous system depends on the expression of specific genes in particular places and periods. Thus, considering the current conditions (adult rat bone marrow MSC and one specific chemical induction protocol), it is very unlikely that, in a few hours of induction, MSC transdifferentiate into mature (or even immature) neurons.

### Conclusion

Our results indicate that after a specific protocol of chemical induction, adult rat derived MSC display some neuronal characteristics (morphology, expression of neural proteins), a number of which can also be seen in other stressed or damaged cells. However, this protocol of chemical MSC induction does not cause these cells to display neuronal electrophysiological properties. Therefore, we suggest that this specific treatment leads to cytoskeletal alterations that are more likely the culprit in cellular stress and cell death. Hence, we conclude that, even though it may be possible to produce neurons from the multipotent cells present in the MSC population, chemically-induced acute transdifferentiation, as used here, is not likely a means to achieve this goal. Nevertheless, it is still plausible that other methods of induction and/or some other sources of MSC are able to achieve a successful neuronal differentiation *in vitro*.

Lastly, we would like to stress the importance of electrophysiological approaches to determine stem cell differentiation into neurons. Morphological changes and protein expression are not conclusive in the stem cell research field and, therefore, the plasticity of stem cells requires careful validation.

## Supporting Information

File S1 Supplemental information about the neuronal primary culture.
(0.03 MB DOC)

Figure S1 Negative controls for the immunocytochemistry method. The primary antibody was lacking to verify the specificity of secondary antibodies. A) Secondary biotinylated antibody anti- mouse IgG; B) Secondary biotinylated antibody anti- rabbit IgG.
(2.64 MB TIF)

## References

[1] Abouelfetouh A, Kondoh T, Ehara K, Kohmura E (2004). Morphological differentiation of bone marrow stromal cells into neuron-like cells after co-culture with hippocampal slice.. Brain Res.

[2] Bonilla S, Silva A, Valdes L, Geijo E, Garcia-Verdugo JM (2005). Functional neural stem cells derived from adult bone marrow.. Neuroscience.

[3] Bossolasco P, Cova L, Calzarossa C, Rimoldi SG, Borsotti C (2005). Neuro-glial differentiation of human bone marrow stem cells in vitro.. Exp Neurol.

[4] Hermann A, Gastl R, Liebau S, Popa MO, Fiedler J (2004). Efficient generation of neural stem cell-like cells from adult human bone marrow stromal cells.. J Cell Sci.

[5] Lei Z, Yongda L, Jun M, Yingyu S, Shaoju Z (2007). Culture and neural differentiation of rat bone marrow mesenchymal stem cells in vitro.. Cell Biol Int.

[6] Sanchez-Ramos J, Song S, Cardozo-Pelaez F, Hazzi C, Stedeford T (2000). Adult bone marrow stromal cells differentiate into neural cells in vitro.. Exp Neurol.

[7] Scintu F, Reali C, Pillai R, Badiali M, Sanna MA (2006). Differentiation of human bone marrow stem cells into cells with a neural phenotype: diverse effects of two specific treatments.. BMC Neurosci.

[8] Suzuki H, Taguchi T, Tanaka H, Kataoka H, Li Z (2004). Neurospheres induced from bone marrow stromal cells are multipotent for differentiation into neuron, astrocyte, and oligodendrocyte phenotypes.. Biochem Biophys Res Commun.

[9] Woodbury D, Schwarz EJ, Prockop DJ, Black IB (2000). Adult rat and human bone marrow stromal cells differentiate into neurons.. J Neurosci Res.

[10] Deng W, Obrocka M, Fischer I, Prockop DJ (2001). In vitro differentiation of human marrow stromal cells into early progenitors of neural cells by conditions that increase intracellular cyclic AMP.. Biochem Biophys Res Commun.

[11] Jin K, Mao XO, Batteur S, Sun Y, Greenberg DA (2003). Induction of neuronal markers in bone marrow cells: differential effects of growth factors and patterns of intracellular expression.. Exp Neurol.

[12] Kabos P, Ehtesham M, Kabosova A, Black KL, Yu JS (2002). Generation of neural progenitor cells from whole adult bone marrow.. Exp Neurol.

[13] Kohyama J, Abe H, Shimazaki T, Koizumi A, Nakashima K (2001). Brain from bone: efficient “meta-differentiation” of marrow stroma-derived mature osteoblasts to neurons with Noggin or a demethylating agent.. Differentiation.

[14] Alexanian AR, Maiman DJ, Kurpad SN, Gennarelli TA (2008). In vitro and in vivo Characterization of Neurally Modified Mesenchymal Stem Cells Induced by Epigenetic Modifiers and Neural Stem Cell Environment.. Stem Cells Dev.

[15] Corti S, Locatelli F, Strazzer S, Salani S, Del Bo R (2002). Modulated generation of neuronal cells from bone marrow by expansion and mobilization of circulating stem cells with in vivo cytokine treatment.. Exp Neurol.

[16] Munoz-Elias G, Marcus AJ, Coyne TM, Woodbury D, Black IB (2004). Adult bone marrow stromal cells in the embryonic brain: engraftment, migration, differentiation, and long-term survival.. J Neurosci.

[17] Azizi SA, Stokes D, Augelli BJ, DiGirolamo C, Prockop DJ (1998). Engraftment and migration of human bone marrow stromal cells implanted in the brains of albino rats–similarities to astrocyte grafts.. Proc Natl Acad Sci U S A.

[18] Brazelton TR, Rossi FM, Keshet GI, Blau HM (2000). From marrow to brain: expression of neuronal phenotypes in adult mice.. Science.

[19] Kopen GC, Prockop DJ, Phinney DG (1999). Marrow stromal cells migrate throughout forebrain and cerebellum, and they differentiate into astrocytes after injection into neonatal mouse brains.. Proc Natl Acad Sci U S A.

[20] Mezey E, Key S, Vogelsang G, Szalayova I, Lange GD (2003). Transplanted bone marrow generates new neurons in human brains.. Proc Natl Acad Sci U S A.

[21] Svendsen CN, Caldwell MA, Ostenfeld T (1999). Human neural stem cells: isolation, expansion and transplantation.. Brain Pathol.

[22] Munoz-Elias G, Woodbury D, Black IB (2003). Marrow stromal cells, mitosis, and neuronal differentiation: stem cell and precursor functions.. Stem Cells.

[23] Gurok U, Steinhoff C, Lipkowitz B, Ropers HH, Scharff C (2004). Gene expression changes in the course of neural progenitor cell differentiation.. J Neurosci.

[24] Hsieh J, Gage FH (2004). Epigenetic control of neural stem cell fate.. Curr Opin Genet Dev.

[25] Taschenberger H, Scheuss V, Neher E (2005). Release kinetics, quantal parameters and their modulation during short-term depression at a developing synapse in the rat CNS.. J Physiol.

[26] Lu P, Blesch A, Tuszynski MH (2004). Induction of bone marrow stromal cells to neurons: differentiation, transdifferentiation, or artifact?. J Neurosci Res.

[27] Neuhuber B, Gallo G, Howard L, Kostura L, Mackay A (2004). Reevaluation of in vitro differentiation protocols for bone marrow stromal cells: disruption of actin cytoskeleton induces rapid morphological changes and mimics neuronal phenotype.. J Neurosci Res.

[28] Krampera M, Marconi S, Pasini A, Galie M, Rigotti G (2007). Induction of neural-like differentiation in human mesenchymal stem cells derived from bone marrow, fat, spleen and thymus.. Bone.

[29] Rismanchi N, Floyd CL, Berman RF, Lyeth BG (2003). Cell death and long-term maintenance of neuron-like state after differentiation of rat bone marrow stromal cells: a comparison of protocols.. Brain Res.

[30] Wenisch S, Trinkaus K, Hild A, Hose D, Heiss C (2006). Immunochemical, ultrastructural and electrophysiological investigations of bone-derived stem cells in the course of neuronal differentiation.. Bone.

[31] Kelly CM, Tyers P, Borg MT, Svendsen CN, Dunnett SB (2005). EGF and FGF-2 responsiveness of rat and mouse neural precursors derived from the embryonic CNS.. Brain Res Bull.

[32] Brewer GJ (1995). Serum-free B27/neurobasal medium supports differentiated growth of neurons from the striatum, substantia nigra, septum, cerebral cortex, cerebellum, and dentate gyrus.. J Neurosci Res.

[33] Pfeiffer CM, Huff DL, Gunter EW (1999). Rapid and accurate HPLC assay for plasma total homocysteine and cysteine in a clinical laboratory setting.. Clin Chem.

[34] Bianco P, Riminucci M, Gronthos S, Robey PG (2001). Bone marrow stromal stem cells: nature, biology, and potential applications.. Stem Cells.

[35] Croft AP, Przyborski SA (2006). Formation of neurons by non-neural adult stem cells: potential mechanism implicates an artifact of growth in culture.. Stem Cells.

[36] Alvarez-Dolado M, Pardal R, Garcia-Verdugo JM, Fike JR, Lee HO (2003). Fusion of bone-marrow-derived cells with Purkinje neurons, cardiomyocytes and hepatocytes.. Nature.

[37] Bae JS, Han HS, Youn DH, Carter JE, Modo M (2007). Bone marrow-derived mesenchymal stem cells promote neuronal networks with functional synaptic transmission after transplantation into mice with neurodegeneration.. Stem Cells.

[38] Goolsby J, Marty MC, Heletz D, Chiappelli J, Tashko G (2003). Hematopoietic progenitors express neural genes.. Proc Natl Acad Sci U S A.

[39] Lu P, Tuszynski MH (2005). Can bone marrow-derived stem cells differentiate into functional neurons?. Exp Neurol.

[40] Ratajczak MZ, Kucia M, Reca R, Majka M, Janowska-Wieczorek A (2004). Stem cell plasticity revisited: CXCR4-positive cells expressing mRNA for early muscle, liver and neural cells ‘hide out’ in the bone marrow.. Leukemia.

[41] Tondreau T, Lagneaux L, Dejeneffe M, Massy M, Mortier C (2004). Bone marrow-derived mesenchymal stem cells already express specific neural proteins before any differentiation.. Differentiation.

[42] Woodbury D, Reynolds K, Black IB (2002). Adult bone marrow stromal stem cells express germline, ectodermal, endodermal, and mesodermal genes prior to neurogenesis.. J Neurosci Res.

[43] Schoenherr CJ, Anderson DJ (1995). The neuron-restrictive silencer factor (NRSF): a coordinate repressor of multiple neuron-specific genes.. Science.

[44] Kuwabara T, Hsieh J, Nakashima K, Taira K, Gage FH (2004). A small modulatory dsRNA specifies the fate of adult neural stem cells.. Cell.

[45] Martin D, Tawadros T, Meylan L, Abderrahmani A, Condorelli DF (2003). Critical role of the transcriptional repressor neuron-restrictive silencer factor in the specific control of connexin36 in insulin-producing cell lines.. J Biol Chem.

[46] Beyer Nardi N, da Silva Meirelles L (2006). Mesenchymal stem cells: isolation, in vitro expansion and characterization.. Handb Exp Pharmacol.

[47] Laterza OF, Modur VR, Crimmins DL, Olander JV, Landt Y (2006). Identification of novel brain biomarkers.. Clin Chem.

[48] Jones DP, Go YM, Anderson CL, Ziegler TR, Kinkade JM (2004). Cysteine/cystine couple is a newly recognized node in the circuitry for biologic redox signaling and control.. Faseb J.

[49] Liu Y, Gutterman DD (2002). Oxidative stress and potassium channel function.. Clin Exp Pharmacol Physiol.

[50] Miller BA (2006). The role of TRP channels in oxidative stress-induced cell death.. J Membr Biol.

[51] Hool LC, Corry B (2007). Redox control of calcium channels: from mechanisms to therapeutic opportunities.. Antioxid Redox Signal.

[52] Sagara JI, Makino N (2007). Glutathione Induces Neuronal Differentiation in Rat Bone Marrow Stromal Cells.. Neurochem Res.

[53] Hille B (2001). Ion Channels of Excitable Membranes..

[54] Goodwin PA, Campbell KE, Evans BA, Wann KT (1998). In vitro patch-clamp studies in skin fibroblasts.. J Pharmacol Toxicol Methods.

[55] Li GR, Deng XL, Sun H, Chung SS, Tse HF (2006). Ion channels in mesenchymal stem cells from rat bone marrow.. Stem Cells.

[56] Lang F, Huber SM, Szabo I, Gulbins E (2007). Plasma membrane ion channels in suicidal cell death.. Arch Biochem Biophys.

[57] Bortner CD, Cidlowski JA (2007). Cell shrinkage and monovalent cation fluxes: role in apoptosis.. Arch Biochem Biophys.

[58] Watanabe M, Sakimura K, Takahashi Y, Kondo H (1990). Ontogenic changes in expression of neuron-specific enolase (NSE) and its mRNA in the Purkinje cells of the rat cerebellum: immunohistochemical and in situ hybridization study.. Brain Res Dev Brain Res.

