# Supplementary material for: Chemically-Induced RAT Mesenchymal Stem Cells Adopt Molecular Properties of Neuronal-Like Cells but Do Not Have Basic Neuronal Functional Properties
Source: PLoS One. 2009 Apr 16;4(4):e5222. doi: 10.1371/journal.pone.0005222 (PMC2667250; doi:10.1371/journal.pone.0005222)
Supplement: File S1 — Supplemental information about the neuronal primary culture. (0.03 MB DOC) [file pone.0005222.s001.doc]

**Supplemental information about the neuronal cultures:** Rat neural precursor cells (NPC) [31] and primary culture of rat fetal neurons from the brain cortex [32] were obtained as described previously [31,32]. Pregnant rats were sacrificed in a CO_2_ chamber, in accordance with institutional guidelines. Telencephalons of E14 and E17 rat fetuses were used to make neural precursor cells and primary neuronal cultures, respectively. Fetuses were dissected and the tissue was incubated with trypsin-EDTA solution (Stem Cell Technologies, Vancouver, BC, Canada) for 10 minutes at 37ºC. After trypsin inactivation cells were mechanically dissociated. Neural precursor cells were cultured in passaging medium [DMEM/F12 (70% DMEM and 30% F12), 1% penicillin / streptomycin / amphotericin, 20 ng/mL EGF (Sigma); 20 ng/mL FGF-2 (R&D Systems, Minneapolis, MN, USA), 5 μg/mL heparin (Sigma) and 2% B27 supplement (Gibco)] and grown as neurospheres. Then, neurospheres were plated onto coverslips coated with poly-L-lysine (Sigma) and laminin (purified from cultured tumor cells) donated by Prof. Helena B. Nader, Department of Biochemistry, Universidade Federal de São Paulo), in differentiation medium (DMEM/F12, 1% penicillin / streptomycin / amphotericin, 2% B27 supplement), allowing them to migrate and differentiate for 7 days. Primary cultures of neurons were dissociated and plated onto poly-L-lysine coated coverslips in DMEM/F12, 1% penicillin / streptomycin / amphotericin and 1% N2 supplement (Gibco). Cells were maintained in an incubator at 37 ºC with a 5% CO_2_ atmosphere and > 95% humidity.
