# Supplementary figures and images for: Chemically-Induced RAT Mesenchymal Stem Cells Adopt Molecular Properties of Neuronal-Like Cells but Do Not Have Basic Neuronal Functional Properties
Source: PLoS One. 2009 Apr 16;4(4):e5222. doi: 10.1371/journal.pone.0005222 (PMC2667250; doi:10.1371/journal.pone.0005222)

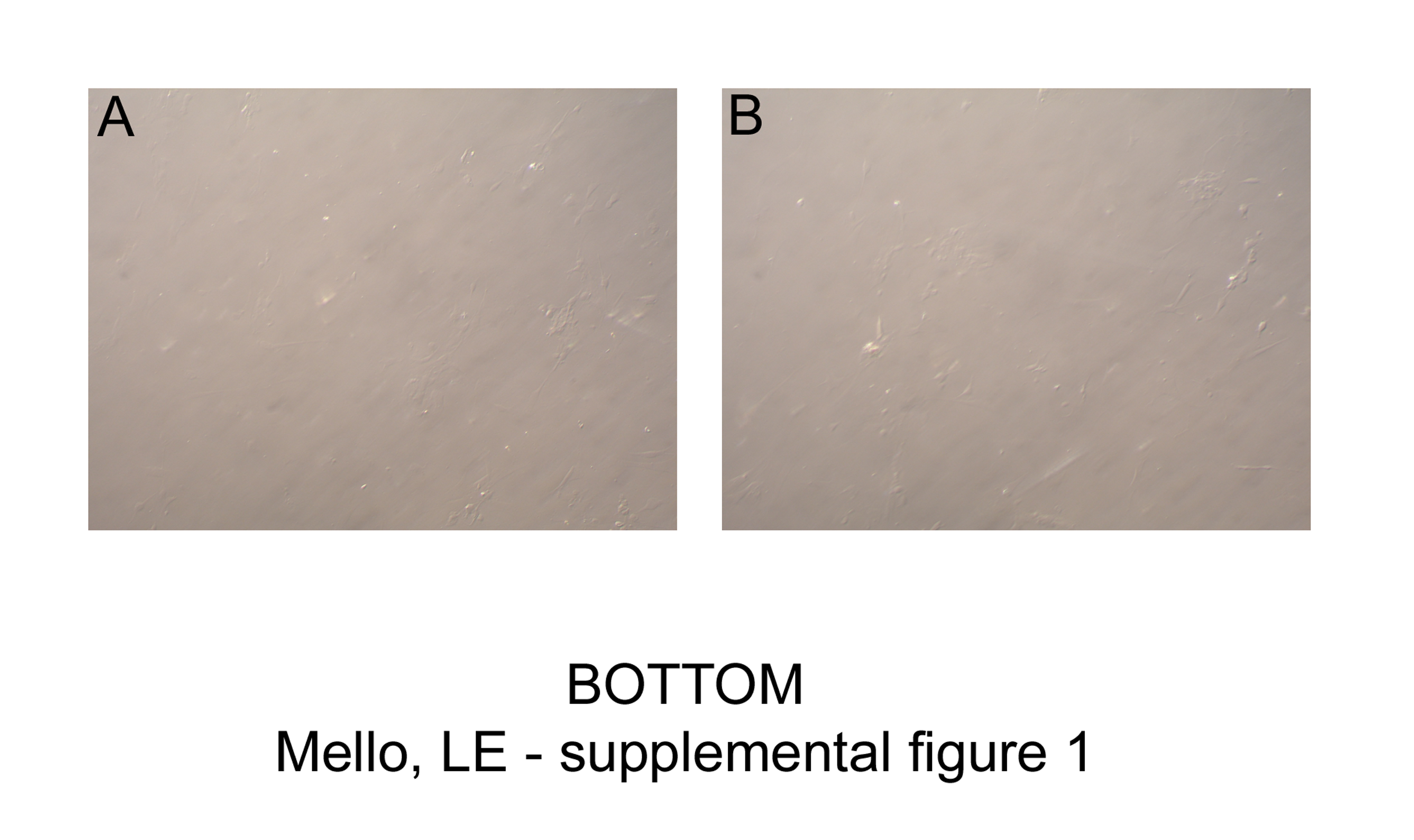

Supplement: Figure S1 — Negative controls for the immunocytochemistry method. The primary antibody was lacking to verify the specificity of secondary antibodies. A) Secondary biotinylated antibody anti- mouse IgG; B) Secondary biotinylated antibody anti- rabbit IgG. (2.64 MB TIF) [file pone.0005222.s002.tif]
